# Sleep disorder experienced by healthcare nurses after terminating Zero-COVID-19 policy

**DOI:** 10.1186/s12912-024-02145-y

**Published:** 2024-07-09

**Authors:** Minyi Su, Mingzhu Feng, Wanling Pan, Xuelan Huang, Lei Pan, Yanling Zhu, Le Wang, Mohammad Mofatteh, Adam A Dmytriw, Dongxia Liang, Shuling Wang, Wanyi Liang, Yu Chen, Yimin Chen, Weiping Yao, Qiubi Tang

**Affiliations:** 1 Department of Nursing, Foshan Sanshui District People’s Hospital, Foshan, Guangdong Province 528100 China; 2 Department of Nursing, First People’s Hospital of Foshan, Foshan, Guangdong Province 528000 China; 3 Department of Neurology and Advanced National Stroke Center, Foshan Sanshui District People’s Hospital, Foshan, Guangdong Province China; 4 Dean Office, Foshan Sanshui District People’s Hospital, Foshan, Guangdong Province China; 5 Epidemic Prevention and Control Team, Foshan Sanshui District People’s Hospital, Foshan, Guangdong Province China; 6 Department of Psychosomatic Medicine, Nanhai Public Health Hospital of Foshan City (Nanhai Mental Health Center), Foshan, Guangdong Province China; 7 School of Medicine, Dentistry and Biomedical Sciences, Queen’s University Belfast, Belfast, UK; 8 Neuroendovascular Program, Massachusetts General Hospital, Harvard Medical School, Boston, MA USA; 9 Department of Psychology, Foshan Sanshui District People’s Hospital, Foshan, Guangdong 528100 China; 10 School of Nursing, Guangdong Medical University, Zhanjiang, China; 11 Fever Clinic, Foshan Sanshui District People’s Hospital, Foshan, Guangdong Province China; 12 Department of Nursing, The Affiliated Brian Hospital of Guangzhou Medical University, Chronic Disease Department, Guangzhou, China

**Keywords:** Nurse, Sleep disorder, Pittsburgh Sleep Quality Index, COVID-19 pandemic

## Abstract

**Objective:**

Medical staff, especially nurses, suffered great anxiety and stress from the COVID-19 pandemic, which negatively affected their sleep quality. In this study, we aimed to analyze the sleep quality of nursing staff after terminating the Zero-COVID-19 policy in China.

**Methods:**

506 participants were involved in our study. The Pittsburgh Sleep Quality Index (PSQI) was used to evaluate the sleep status of the participants. Binary regression was performed to evaluate the impact factors related to sleep difficulty.

**Results:**

The majority of participants (96.44%) suffered from sleep disturbances. There were significant differences in age, education level and front-line activity between participants with good sleep quality and sleep difficulty. Younger age (16–25 years old) was independently associated with less sleep difficulty, while front-line activity was independently associated with severe sleep difficulty.

**Conclusion:**

Sleep disorder was very common among nurses after ending the Zero-COVID-19 policy in China. More front-line nurses suffered severe sleep difficulty in particular, which should be worthy of attention.

## Introduction

The global spread of corona virus disease 2019 (COVID-19) has become a major public health event [1]. The COVID-19 pandemic resulted in an unprecedented challenge for society, especially in the healthcare sector [2]. This situation has entailed great responsibility for health workers, translating into both physical and psychological high pressure due to their exposure to the disease with a fear of contamination and the extra effort required to adapt their usual work routine to the new hospital conditions caused by the pandemic, such as wearing personal protective equipment, attending extremely ill patients, working irregular hours [3]. On 2022 November, the Chinese government optimized the epidemic prevention and control policy, and ending ‘Zero-COVID-19’ policy and mandatory quarantine measures [4]. This new strategy caused a swift and extensive outbreak of the Omicron variant in China after a sudden exit from the Zero-COVID-19 policy [5]. A surge in the number of patients visiting healthcare facilities introduced additional pressure on healthcare workers in an already strained healthcare system [6]. After terminating the policy, hospitals in China faced a sudden increase in patients presenting with fever and/or respiratory diseases in a short period of time. Healthcare staff dealt with the situation by converting regular care beds to severe care beds in a timely manner, and suffered great pressure [7].

Multiple studies reported that psychological and physical stressors among nurses were common during the pandemic [8]. Psychological and physical stress can cause long-term sleep deprivation leading to severe fatigue, memory loss, slow response, irritability, depression, suicidal ideation and occupational accidents [9]. Therefore, improving sleep quality is crucial, especially among healthcare workers, to improve their mental health, job satisfaction and ultimately delivering the best patient care possible [10].

A systematic review and meta-analysis showed that about one-third of nurses during the COVID-19 pandemic had psychological symptoms such as stress, anxiety, depression, and sleep disorders. The incidence of sleep disorders is 43% [11]. Another meta-analysis reported that the prevalence of sleep disorders among healthcare workers during the COVID-19 pandemic was 38.27% [12]. Although these meta-analyses highlight the poor sleep quality of nurses or healthcare workers during the pandemic, to date, there is currently very little research on the sleep quality of nurses in China after the end of the Zero COVID-19 policy. Therefore, the aim of this study was to evaluate the sleep disorder of nurses after China terminated Zero-COVID-19 policy.

## Methods

### Clinical characteristic and data collection

A total of 506 nurses engaged in COVID-19-related nursing tasks in Foshan Sanshui District People’s Hospital participated in the study from December 2022 to January 2023 by responding to a questionnaire. Investigators explained the purpose, significance, and relevant instructions of the study before conducting the survey, and signed consent forms were obtained. The first section of the questionnaire collected social demographic information, including department, concern to study, age, marital status, and highest educational degree obtained. The second section included the Pittsburg Sleep Quality Index scale (PSQI). The questionnaire was distributed by https://www.wjx.cn via WeChat (a popular Chinese communication application) from December 2022 to January 2023.

### Population/sample

All nurses working during the study period (including frontline and non frontline nurses) were invited to participate. Nurses answered the questionnaire and accepted conditional inclusion in informed consent. A total of 506 questionnaires were distributed and 506 valid questionnaires were collected. No questionnaire data is missing, so all responses to the questionnaire are valid and included in this study.

### Assessment of sleep status

PSQI can identify participants with good sleep or sleep difficulty [13]. It is a administered questionnaire with 19 questions and includes seven sections: subjective sleep quality, sleep latency, sleep duration, sleep efficiency, sleep disturbances, use of hypnotic medication and daytime dysfunction. Each part scores from 0 to 3, and the total score ranks from 0 to 21, scores of ≤ 5 were considered good sleepers, whereas scores ≥ 6 were considered poor sleepers [13]. Sleep disorder can be divided by scores: 0–5 is a good sleep score; 6–10 shows mild sleep difficulty; 11–15 moderate sleep difficulty; and 16–21 severe sleep difficulty [14]; and scores of 0–15 was considered not severe sleep difficulty.

### Definition of front-line nurses

Nurses working in the emergency department, pneumology department, infectious department, intensive care unit, and fever clinic were impacted seriously by the COVID-19 pandemic and, therefore, were considered as front-line nurses.

### Data analysis

Statistical analysis was performed using the SPSS Statistics Package (Version 26.0; IBM Corporation, Armonk, NY, USA). Proportions were expressed in percentages. the chi-square (χ2) test, Continuity Correction or Fisher's Exact Test for proportions according to requirements. Multivariate binary logistics regression was performed to further analyze for independent factors. A *p*-value of < 0.05 was considered statistically significant.

## Results

### Participants’ characteristics

Participants’ general demographics are shown in Table 1. There were 506 Participants in the study. The age of the participating nurses ranged from 16 to 55 years. Nurses aged between 16 to 35 mostly participated in the study (16–25 years old: 28.46% versus 26–35 years old: 28.46%). Those aged 36 to 45 years had the next highest participation rate of 19.17%. The oldest age group (46–55) had the least participation at about 8.70%. 30.24% of the participants were frontline nurses. The majority of the participants were females (94.27%), and more than half of the participants (55.93%) were married. 61.66% of the participants had a Bachelor’s degree.

**Table 1:** Participants general demographics

Variable	N (%)
Age (year)	
16–25	144 (28.46)
26–35	221 (28.46)
36–45	97 (19.17)
46–55	44 (8.70)
Department	
Frontline nurse	153 (30.24)
Not Frontline nurse	353 (69.76)
Sex	
Male	29 (5.73)
Female	477 (94.27)
Marital status	
Married	283 (55.93)
Single	210 (41.50)
Divorced	12 (2.37)
Widowed or others	1 (0.20)
Highest degree	
Associate degree or below	192 (37.94)
Bachelor’s degree	312 (61.66)
Postgraduate or higher	2 (0.40)

### Sleep status

Only 3.56% of participants reported having good sleep quality. Most participants (96.44%) suffered from sleep disturbances, with 33.00% having mild sleep disturbances, 42.09% having moderate sleep disturbances, and 21.34% having severe sleep disturbances (Table 2).

**Table 2:** Participants sleep status

Sleep status	n, %
Good sleep	18 (3.56)
Mild sleep difficulty	167 (33.00)
Moderate sleep difficulty	213 (42.09)
Severe sleep difficulty	108 (21.34)
Sleep difficulty	488 (96.44)

### Comparison of impact factors between good sleep and sleep difficulty participants

Statistically significant differences were found in age and highest education level between nurses with good sleep quality and those with sleep difficulty (*p* < 0.05). Most participants (61.11%) with good sleep quality were nurses aged 16 to 25. Among those with sleep difficulty, 26 to 35 years old accounted for the largest proportion (44.67%). Participants with good sleep quality (72.22%) generally had a low education level, with only an associate degree or below. 62.91% of participants with a Bachelor's degree had sleep difficulty. No significant differences were observed in sex, marital status and front-line activity of nurses (Table 3).

**Table 3:** Good sleep vs sleep difficulty

Variable		Good sleep *N* = 18	Sleep difficulty *N* = 488	χ^2^	*p*
Sex	Male	1 (5.56)	28 (5.74)	0.000	1.000
	Female	17 (94.44)	460 (94.26)		
Age (years)	16–25	11 (61.11)	133 (27.25)	9.774	0.002^**^
Age (years)	26–35	3 (16.67)	218 (44.67)	5.535	0.019^*^
Age (years)	36–45	3 (16.67)	94 (19.26)	0.075	0.784
Age (years)	46–55	1 (5.56)	43 (8.81)	0.003	0.956
Marital status	Married	7 (38.89)	276 (56.56)	N/A	0.249
	Single	11 (61.11)	199 (40.78)		
	Divorced	0 (0.00)	12 (2.46)		
	Widowed or others	0 (0.00)	1 (0.20)		
Highest education	Associate degree or below	13 (72.22)	179 (36.68)	9.313	0.002^**^
Highest education	Bachelor’s degree	5 (27.78)	307 (62.91)	9.063	0.003^**^
Highest education	Postgraduate or higher	0 (0.00)	2 (0.41)	N/A	1.00
Frontline Nurses		8 (44.44)	145 (29.71)	1.786	0.181

^*^p < 0.05

^**^p < 0.01

### Comparison of impact factors between participants without severe sleep difficulty and those severe sleep difficulty

Statistically significant differences were found in age and front-line nurses between those without severe sleep difficulty and those with severe sleep difficulty (*p* < 0.05). Younger participants aged 16–25 years had less severe sleep difficulty (*p* = 0.001), whereas older participants aged 46–55 years tended to have more severe sleep difficulty, but it did not reach a statistical significance level (*p* = 0.076). The front-line nurses were associated with severe sleep difficulty (*p* = 0.004). No significant differences were observed in other variables (Table 4).

**Table 4:** Factors of severe sleep difficulty

Varies		Not severe sleep difficulty (0–15) *N* = 298	Severe sleep difficulty (16–21) *N* = 108	χ^2^	*p*
Sex	Male	21 (5.28)	8 (7.41)	0.714	0.398
	Female	377 (94.72)	100 (92.59)		
Age	16–25	127 (31.91)	17 (15.74)	10.908	0.001^**^
Age	26–35	167 (41.96)	54 (50.00)	2.232	0.135
Age	36–45	74 (18.59)	23 (21.30)	0.401	0.527
Age	46–55	30 (7.54)	14 (12.96)	3.149	0.076
Marital status	Married	219 (55.03)	64 (59.26)	N/A	0.466
	Single	170 (42.71)	40 (37.04)		
	divorce	8 (2.01)	4 (3.70)		
	Widowed or others	1 (0.25)	0 (0.00)		
Highest Degree	associate degree or below	155 (38.94)	37 (34.26)	N/A	0.281
	Bachelor’s degree	242 (60.80)	70 (64.81)		
	Postgraduate or higher	1 (0.25)	1 (0.93)		
Frontline Nurses		108 (27.14)	45 (41.67)	8.503	0.004^**^

^*^^*^p < 0.01

### Factors of severe sleep difficulty by binary regression

Binary regression showed that younger age (16–25 year old) was associated with less severe sleep difficulty as an independent factor, but the front-line nurses were associated with severe sleep difficulty independently (Table 5).

**Table 5:** Factors of severe sleep difficulty by binary regression

	Coefficient	Standard error	*z*	Wald χ^2^	*p*	OR	95% CI
Age of (16–25)	-0.941	0.294	-3.203	10.256	0.001	0.390	0.220 ~ 0.694
Frontline-Nurse	0.793	0.234	3.383	11.447	0.001	2.210	1.396 ~ 3.499
Age of 46–55	0.552	0.357	1.546	2.392	0.122	1.737	0.863 ~ 3.499

## Discussion

The present study showed that sleep disorder was prevalent among nurses after the Zero-COVID-19 policy adjustment. Additionally, we found a higher risk of sleep difficulty among older age nurses and a lower risk of sleep disturbances among those with lower education levels. More importantly, our study indicated that sleep disorders are more common in nurses working on the front-line. As revealed in this study, 96.44% of participants had sleep difficulty, which demonstrates that sleep disorder is quite common among nurses after the Zero-COVID-19 policy adjustment. This finding was higher than the result of 42.9% reported by Yang [15] and the result of 30% reported by Sun [16] during a non-pandemic period in China. This may be related to the greater anxiety and depression experienced by nurses as they were under heavy workload, and they were at risk of being infected after the government’s adjusting the Zero-COVID-19 policy and mandatory quarantine measures. Besides, a meta-analysis  [12]  showed that the prevalence of sleep disorders among healthcare workers during the COVID-19 pandemic was 38.27% and in another study, the prevalence was 11.3% [17]. There are differences in the summarized prevalence rates among different studies, which can be explained by the diversity of assessment scales, healthcare systems, demographic characteristics, and lifestyles. In addition, another meta-analysis consisting of 93 cross-sectional studies involving a total of 93,112 nurses assessed for the first time that the overall prevalence of sleep disorders among nurses during the COVID-19 pandemic was 43% [11]. It is obvious that after the termination of the Zero-COVID-19 policy, the incidence of sleep disorders among nurses has significantly increased, whether compared with the non-epidemic period or during the COVID-19 pandemic period. This further indicates that terminating the Zero-COVID-19 policy has had a serious negative impact on the sleep quality of nurses.

Previous studies showed that regardless of their place of work, healthcare workers, mainly female nurses, experienced symptoms of anxiety and stress, which resulted in decreased sleep and quality of life [18]. Consistent with a previous study, we found that the front-line nurses were associated with severe sleep difficulty as they have a high risk of infection due to their heavy workload and long working hours [19]. A study clearly demonstrated high rates of stress, anxiety, and depression among front-line healthcare workers caring for COVID-19 patients [20].

Contrary to a previous study, which showed no significant association between age and insomnia [21], our study revealed that nurses with older age (46–55 years old) were more susceptible to sleep disorders. Our finding is similar to those of a 2007 survey of the general population in Brazil, finding that people aged 35 years or older appeared to experience higher levels of sleep disturbance [22]. Sleep problems have become an accepted normal change in sleep physiology with aging [23]. Older nurses were either in menopausal transition or menopausal, suffering from vasomotor instability and psychological symptoms due to changes in estrogen levels [24]. Therefore, the mechanism of neurobiological changes may explain the poor sleep quality of elderly head nurses. In addition, compared with young nurses, older nurses may bear more family responsibilities and economic burdens and are more likely to suffer from negative life events such as divorce and chronic physical diseases [25].

We found that nurses with lower education levels had better sleep quality; however, some cases showed lower levels of education may contribute to insomnia [26]. Nurses with a higher level of education may be more knowledgeable about the effects of COVID-19 on health, and they may be more frequently exposed to occupational stressors, which puts them at risk for developing psychiatric illnesses, leading to sleep difficulty. The current study is the first to compare the effect of nurses' different education levels on sleep quality during the COVID-19 epidemic. More attention should be paid to the education level of nurses affecting sleep quality. Findings from the current study, along with other investigations on the effect of the COVID-19 pandemic on the healthcare system, can be used to identify caveats and improve health provision [27, 28].

Our study has some limitations. First, we relied on self-reported symptoms collected through online questionnaires. While convenient, this approach may be susceptible to bias and inaccuracies inherent in self-reporting. Second, due to the pandemic, we were unable to utilize objective diagnostic tools for sleep disorders. Future studies could benefit from incorporating polysomnography for a more comprehensive assessment. Third, the study design employed a limited number of demographic variables and focused on nurses from a single healthcare center in China. This limits the generalizability of our findings. Future research should consider including a more diverse sample and data collection across multiple centers. While this study utilized age groups for data collection, it is acknowledged that using exact ages might provide a more granular analysis for future research. Finally, we recognize that the busy schedules of nurses may have influenced the time spent completing the questionnaires, potentially impacting data accuracy.

## Conclusion

Sleep disorder was very common after the end of the Zero-COVID-19 policy in China. More frontline nurses suffered severe sleep difficulty in particular. The physical and mental health of nurses, including sleep disorders, is worthy of attention to help improve the quality of life among healthcare.

## Data Availability

The data are available from the corresponding author for reasonable request.
